# Optimizing the Performance of Low-Loaded Electrodes for CO_2_-to-CO Conversion Directly from Capture Medium: A Comprehensive Parameter Analysis

**DOI:** 10.3390/nano13162314

**Published:** 2023-08-12

**Authors:** Alessio Mezza, Mattia Bartoli, Angelica Chiodoni, Juqin Zeng, Candido F. Pirri, Adriano Sacco

**Affiliations:** 1 Center for Sustainable Future Technologies @Polito, Istituto Italiano di Tecnologia, Via Livorno 60, 10144 Torino, Italy; mattia.bartoli@iit.it (M.B.); angelica.chiodoni@iit.it (A.C.); juqin.zeng@polito.it (J.Z.); fabrizio.pirri@polito.it (C.F.P.); 2 Department of Applied Science and Technology, Politecnico di Torino, Corso Duca degli Abruzzi 24, 10129 Torino, Italy

**Keywords:** carbon capture and utilization, CO_2_ valorization, bicarbonate electrolyzer, electrochemical impedance spectroscopy

## Abstract

Gas-fed reactors for CO_2_ reduction processes are a solid technology to mitigate CO_2_ accumulation in the atmosphere. However, since it is necessary to feed them with a pure CO_2_ stream, a highly energy-demanding process is required to separate CO_2_ from the flue gasses. Recently introduced bicarbonate zero-gap flow reactors are a valid solution to integrate carbon capture and valorization, with them being able to convert the CO_2_ capture medium (i.e., the bicarbonate solution) into added-value chemicals, such as CO, thus avoiding this expensive separation process. We report here a study on the influence of the electrode structure on the performance of a bicarbonate reactor in terms of Faradaic efficiency, activity, and CO_2_ utilization. In particular, the effect of catalyst mass loading and electrode permeability on bicarbonate electrolysis was investigated by exploiting three commercial carbon supports, and the results obtained were deepened via electrochemical impedance spectroscopy, which is introduced for the first time in the field of bicarbonate electrolyzers. As an outcome of the study, a novel low-loaded silver-based electrode fabricated via the sputtering deposition technique is proposed. The silver mass loading was optimized by increasing it from 116 μg/cm^2^ to 565 μg/cm^2^, thereby obtaining an important enhancement in selectivity (from 55% to 77%) and activity, while a further rise to 1.13 mg/cm^2^ did not provide significant improvements. The tremendous effect of the electrode permeability on activity and proficiency in releasing CO_2_ from the bicarbonate solution was shown. Hence, an increase in electrode permeability doubled the activity and boosted the production of in situ CO_2_ by 40%. The optimized Ag-electrode provided Faradaic efficiencies for CO close to 80% at a cell voltage of 3 V and under ambient conditions, with silver loading of 565 μg/cm^2^, the lowest value ever reported in the literature so far.

## 1. Introduction

During the previous decades, human activities have increased atmospheric CO_2_ concentrations, stimulating the scientific community toward the development of less-carbon intensive technologies and depleting the use of fossil fuels. However, renewable energy use requires time to be assessed, and it is of capital relevance to reduce carbon emissions during the energy transition. As a consequence, the renewable-energy-powered electrochemical reduction reaction of CO_2_ (eCO_2_RR) into added-value chemicals and fuels (e.g., syngas and methane) has attracted strong interest as a solution for a close carbon cycle [1]. The possibility to obtain carbon-based products from eCO_2_RR at high rates has already been deeply investigated in gas-fed electrolyzers [2], where a stream of pure CO_2_ needs to be delivered to the cathode. The perspective to employ such technology in an industrial setting requires coupling between the electrolyzer and CO_2_ separation from the other components of flue gasses (e.g., O_2_, N_2_, and H_2_O) emitted by a point source (e.g., an industrial plant). As an example, alkaline solutions (e.g., KOH) are able to capture gaseous CO_2_ from flue gasses thanks to reactions that form (bi)carbonates [3]. Since it is known that CO_2_ may be extracted from bicarbonate through energy-intensive processes [4], once it has been pressurized, it can be exploited for further valorization through electrolytic conversion. [5] In such kinds of platforms for carbon capture and utilization (CCU), since common gas-fed electrolyzers exhibit low single-pass utilization, around 80% of delivered CO_2_ exits from the platform as unreacted gas [6]. In this framework, liquid-fed bicarbonate (HCO_3_^−^) electrolyzers have arisen as a new, groundbreaking technology to integrate the capture and conversion of CO_2_ (ICCU) [7,8,9] into CO. These reactors introduced the chance to eliminate all the energy-demanding processes (capture/stripping and pressurization) necessary to feed a classical gas-fed eCO_2_RR system. The electrolysis of carbonate solution, i.e., the capture media, is possible using a cation exchange membrane (CEM) or a bipolar membrane (BPM) that, providing an acidic local environment, makes gaseous CO_2_ available in proximity to the catalyst for electroreduction. The utilization of a BPM instead of a CEM is now benchmarked since it allows for the employment of an inexpensive nickel anode and prevents products’ cross-over [10,11]. The BPM, together with anodic and cathodic catalysts, constitutes a membrane electrode assembly (MEA), which is the benchmark configuration in bicarbonate electrolyzers. In the MEA, the presence of H^+^ (produced in the BPM by water splitting) at the membrane/catalyst interface is responsible for the in situ acidification and thus extraction of CO_2_ (*i*-CO_2_) from (bi)carbonate, which is converted into CO (Equations (1) and (2)), thanks to the eCO_2_RR catalyst.

H^+^ + HCO_3_^−^ ↔ H_2_O + CO_2_ (g) (1)

CO_2_(g) + H_2_O + 2e^−^ → 2OH^−^ + CO (2)

Since OH^−^ is a product of the CO_2_RR as well, the original alkaline capture solution is regenerated, making this system able to implement a closed cycle where CO_2_ is sequentially captured and converted. The presence of a MEA ensures a very high local concentration of CO_2_ at the electrocatalyst interface without the need of supplying the reactor by a stream of gaseous CO_2_ in stoichiometric excess, as happens with gas-fed electrolyzers. This also means that CO_2_RR products are generated at higher concentrations [12]. The gas diffusion electrode (GDE) employed in this kind of system has to ensure the efficient transport of carbon feedstock (i.e., HCO_3_^−^) at the BPM/catalyst interface. Therefore it must be engineered differently with respect to the GDEs used in gas-fed electrolyzers, which usually exhibit hydrophobic properties to avoid the accumulation of water and to mitigate the hydrogen evolution reaction (HER) [13], the competing reaction of the CO_2_RR.

Despite the promising advantages, research conducted on this technology so far is still limited compared to more well-known gas-fed electrolyzers. Therefore, a deep investigation into every aspect of the system is needed. Among the first works, Y. C. Li et al. [8] reported a bicarbonate electrolyzer able to keep the high pH of the capture solution for 145 h by using a carbon composite silver electrode, but the highest Faradaic efficiency (FE) toward CO (FE_CO_) was ~35%. T. Li et al. [7] with a silver nanoparticle-coated carbon support obtained impressive FE_CO_ at a low current density and showed how the employment of an anion exchange membrane (AEM) is detrimental to the electrolyzer’s performance. The same conclusion was reported by C. Larrea et al. [14], whereby, although it was responsible for a large ohmic drop between the two electrodes, the necessity to use a BPM in order to have appreciable FE was proven. Z. Zhang et al. [15] showed how the increase in porosity of a silver foam, employed as a cathode, enables more efficient CO_2_ conversion; however, even avoiding the utilization of composite carbon electrodes, the FE_CO_ achieved at ambient conditions is around 60%. In addition, they illustrated how higher pressure and higher temperature promote the CO_2_RR. Y. Kim et al. [16] underlined the importance of a trade-off between the active surface and the permeability of the GDE in order to guarantee both a high CO_2_RR rate and efficient transport of bicarbonate (i.e., *i*-CO_2_ generation). E. W. Lees et al. [17] reported important information on the spray coating of a silver catalyst in order to have an efficient GDE in terms of Nafion content and Ag nanoparticle loading. By adding a preliminary deposition step by sputtering physical vapor deposition (PVD) before the spray coating, they reached a very good FE_CO_ (around 82%) using a high silver loading of 2 mg/cm^2^.

The sputtering technique serves as a rapid and reproducible method for manufacturing nanostructured Ag-GDEs with a high surface area in a single step, offering precise control over catalyst loading, layer thickness, and homogeneity. Our research group has already explored and established the reliability of this approach [18]. In this study, we further optimized the sputtering process to fabricate a GDE specifically designed for bicarbonate electrolyzers. To investigate the electrode’s performance, we tested different commercial carbon supports with distinct characteristics such as gas diffusion layers (GDLs). This allowed us to delve into the GDL’s role and its impact on the FE_CO_ and CO_2_ utilization, representing the extent of CO_2_ conversion compared to the unreacted CO_2_. During the analysis, we explored the influence of several structural and morphological properties of the cathode on the electrochemical performance. These properties encompassed different catalyst distributions on the GDL, GDL hydrophobicity and permeability. By understanding the significance of these factors, we gain critical insights into optimizing the GDE’s design and performance for bicarbonate electrolyzers.

Moreover, electrochemical impedance spectroscopy (EIS) has already demonstrated its efficacy as an efficient tool for studying the charge transfer and transport processes involved in typical systems for CO_2_ reduction reactions (CO_2_RRs) [19]. Its utility has made it a valuable technique for the characterization of materials and reactors in this field [20,21]. Despite this, to the best of our knowledge, it has never been employed in studies involving bicarbonate electrolyzers. Remarkably, our paper presents a pioneering application of EIS to investigate the performance of GDEs employed in bicarbonate electrolyzers. This novel approach represents a significant advancement in the field, as previous research has primarily focused on using EIS for other CO_2_RR systems. Through careful modeling of the GDE/electrolyte interface using an equivalent electrical circuit, this paper successfully elucidates the underlying factors influencing the activity and Faradaic efficiency trends of the GDEs.

## 2. Materials and Methods

### 2.1. Preparation and Morphological Characterization of Ag GDEs

DC Sputtering (Quorum Technologies Ltd., Lewes, UK, Q150T) was used to prepare the Ag electrodes. Three commercial carbon papers (GDL, Ion Power) of 5 cm^2^ characterized by different permeability and wettability (Table S1) were used as substrates, with a silver disk (99.999%, Nanovision, Brugherio, Italy) as the target. The deposition current was fixed at 50 mA, while the deposition time was varied to control the silver mass-loading (100 s, 300 s, and 600 s). A total of 6 GDE samples (A–F) were prepared, whose properties are reported in Table S2. All of the samples were prepared by depositing silver on both faces of the carbon papers, except for sample A. The mass loading was determined by weighing the sample before and after the silver deposition and then by dividing the weight difference by the geometric area of the GDL. The morphology of the commercial carbon-based supports and Ag-GDE samples was investigated by field emission scanning electron microscopy (FESEM, Zeiss Auriga, Oberkochen, Germany).

### 2.2. Electrochemical Tests and Product Analyses

**Figure 1:**
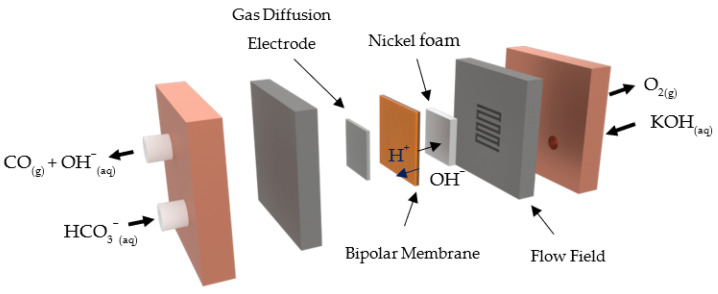
Schematic representation of the bicarbonate MEA electrolyzer.

The electrochemical screening was performed in a bicarbonate electrolyzer (Scribner, Cell Fixture) placed in a vertical position, whose schematic representation is reported in Figure 1. A more detailed description of the system is provided in the Supporting Information. A 5 cm^2^ MEA was employed in the electrolyzer, and it was made by a bipolar membrane (FumaSep FBM, FumaTech, Bietigheim-Bissingen, Germany) sandwiched between a Nickel foam (99.5%, GoodFellow, Huntingdon, UK) and a Ag GDE. KHCO_3_ (99.5%, Sigma-Aldrich, St. Louis, MO, USA) 2 M was used as a catholyte and KOH (Sigma-Aldrich) 1 M was used as an anolyte by dissolving 200 g and 56 g, respectively, in 1 L of ultra-pure water. A peristaltic pump was used to continuously recirculate 60 mL of bicarbonate solution and 40 mL of potassium hydroxide at a flow rate of 5 mL/min. Electrolysis was carried out at ambient temperature and pressure by applying a constant cell voltage (V_cell_) of 3 V (Potentiostat, BioLogic VSP, Seyssinet-Pariset, France). Gas-phase products were delivered to a microgas chromatograph (μGC, Fusion, INFICON) by a N_2_ 35 mL/min stream (Bronkhorst, EL-FLOW select) and analyzed on-line throughout the entire duration of the experiment. The microgas chromatograph, which is preceded by a mass flow reader (Bronkhorst, Ruurlo, The Netherlands, EL-FLOW prestige), is composed of two channels with a 10 m Rt-Molsieve 5A column and an 8 m Rt-Q-Bond column, and each channel has a microthermal conductivity detector. Two tests were conducted per set of experiments, and the results are reported as average values (the error bars correspond to the absolute error). Additional details on the calculation of the CO partial current density, FE, CO_2_ utilization, mass activity, and partial mass activity are provided in the Supporting Information.

### 2.3. Electrochemical Impedance Spectroscopy

EIS measurements were performed in a three-electrode single compartment cell at room temperature with a Biologic VSP electrochemical workstation. The working electrode was a Ag GDE with a geometric area of 0.4 cm^2^. A Pt wire was used as the counter electrode, and Ag/AgCl (3 M Cl^−^) was used as the reference, with both purchased from ALS. The electrolyte was a CO_2_-saturated 2 M KHCO_3_ (99.5%, Sigma-Aldrich) aqueous solution. The analysis was performed at a potential of −1 V vs. a reversible hydrogen electrode (RHE) with an AC signal with 10 mV of amplitude and a 0.1–10^5^ Hz frequency range.

## 3. Results and Discussion

**Figure 2:**
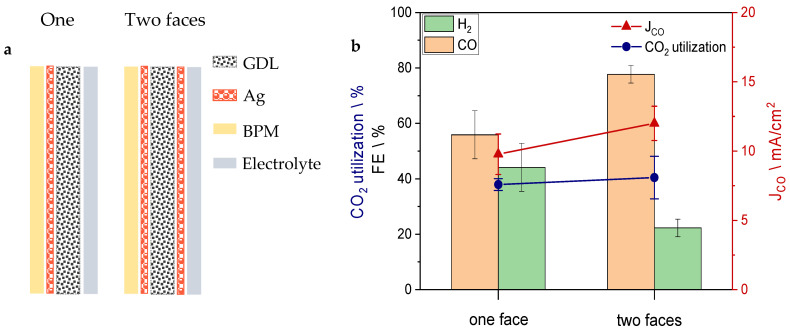
(**a**) Schematic representation of the GDEs with silver sputtered on one face (sample A) and both (sample B) faces. (**b**) FE, CO_2_ utilization and CO partial current density obtained by the two GDEs samples.

As the first step of GDE optimization, it was investigated as to whether it is more convenient to deposit the silver only on one side of the carbon support or on both of them (Figure 2a). Therefore, keeping the same sputtering parameters and carbon support, two samples were made. On the first one (sample A), silver was sputtered only on the face in contact with the bipolar membrane, while on the second one, the sputtering process was replicated on the opposite side as well (sample B), the one facing the graphite flow field. As shown in Figure 2b, sample A exhibits relatively good activity and FE_CO_. This implies that the most active interface is the one facing the bipolar membrane, namely the region with the highest concentration of *i*-CO_2_, since it is in proximity to the BPM. However, sputtering the silver on the other side of the GDL as well boosted the FE_CO_ from 55% to 77%. Considering the results of this experiment, silver was deposited on both faces of the GDE samples tested from then on.

**Figure 3:**
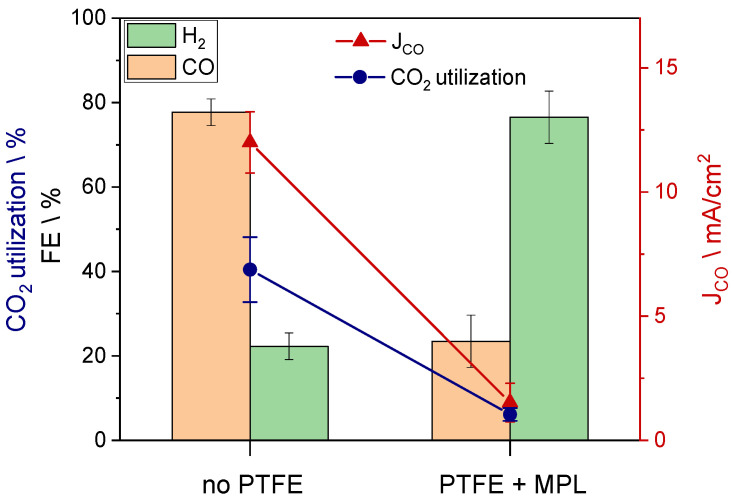
FE, CO_2_ utilization, and CO partial current density obtained with GDEs with two different GDLs: one has not been treated with PTFE and does not include an MPL (sample B), while the other has a strong hydrophobic feature thanks to the PTFE and MPL (sample C).

The carbon support used to obtain the results mentioned just above was not treated with polytetrafluoroethylene (PTFE) nor did it include a microporous layer (MPL). These two characteristics are fundamental if this electrode had been used in a common gas-fed CO_2_RR reactor [18]. In gas-fed reactors, the MPL and the hydrophobic treatment produce water repellent properties that can prevent the carbon fiber backing from flooding. In bicarbonate electrolyzers, the hydrophobic feature inhibits the transport of bicarbonate from the flow field toward the BPM, where the low-pH region is located. In this way, the production of *i*-CO_2_ drastically decreases, hence also the FE_CO_, J_CO_, and the CO_2_ utilization (Figure 3). By using a hydrophobic carbon support (sample C), the FE_CO_ decreases to 23%, while J_CO_ and the overall activity (J_tot_) (Figure S1a) are significantly affected as well.

**Figure 4:**
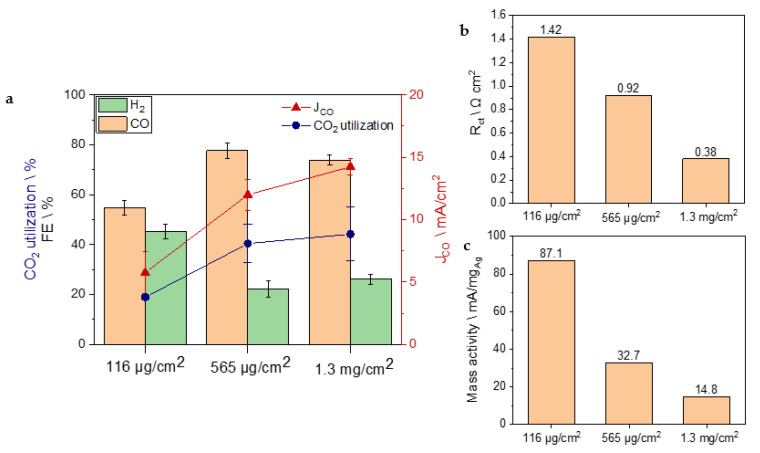
(**a**) FE, CO_2_ utilization, and CO partial current density for different silver mass-loadings: 116 μg/cm^2^ (sample D), 565 μg/cm^2^ (sample B), and 1.13 mg/cm^2^ (sample E). (**b**) Values of R_ct_ that emerged from the EIS analysis. (**c**) Values of mass activity for each sample.

Once the importance of using a GDE with no hydrophobic treatment and presence of a catalyst on both its faces has been confirmed, the silver mass loading was optimized by modulating the sputtering time. The performances of GDEs with silver mass loading of 116 μg/cm^2^ (sample D), 565 μg/cm^2^ (sample B), and 1.13 mg/cm^2^ (sample E) were explored by carrying out electrolysis in the flow cell and an EIS analysis in a three-electrodes set-up. As reported in Figure 4a, the sample with 116 μg/cm^2^ of silver shows the lowest FE (55%), while the other two samples with higher mass loading exhibit better selectivity toward CO. However, since the 1.13 mg/cm^2^ sample did not provided any improvement in selectivity with respect to 565 μg/cm^2^, the latter was identified as optimal catalyst loading since it achieved an FE_CO_ value of 77%, namely, to the best of our knowledge, the lowest loaded silver-based GDE reported in the literature so far (Table 1). Most probably, the great amount of material deposited in sample E lowered the permeability of the GDE, inhibiting the mass transport of bicarbonate and affecting the selectivity. The performance in terms of CO_2_ utilization followed a similar trend to the Faradaic efficiency: it was doubled by increasing the silver loading from 116 μg/cm^2^ to 565 μg/cm^2^, while, with sample E, the increase to 1.13 mg/cm^2^ of silver loading did not further enhance the CO_2_ utilization. J_CO_ and J_tot_ (Figure S1a) increased with higher loadings as confirmed by the EIS analysis (Table S3 and Figure S2). Indeed, the increasing trend of activities observed during bicarbonate electrolysis could be related to the value of the charge transfer resistance (R_ct_). This parameter describes the catalyst’s ability to exchange electrons with the reactants, applicable to both the CO_2_RR and HER. R_ct_ decreases from 1.42 Ω cm^2^ to 0.92 Ω cm^2^ when augmenting the amount of silver from 116 μg/cm^2^ to 565 μg/cm^2^ (Figure 4b). A further decrease (0.38 Ω cm^2^) was experienced with the highest loaded sample (E). Since the electrochemical surface area (ECSA) is considered to be proportionally associated to the double layer capacitance C_dl_ derivable from the EIS analysis (Table S3) [22], the intrinsic activity of various materials can be compared by investigating the C_dl_ normalized current densities (Figure S3) [23]. This investigation confirmed 565 μg/cm^2^ as the optimal mass loading since it showed the highest C_dl_ normalized current density, hence the largest presence of active sites for the CO_2_RR to CO. However, the higher mass activity obtained with sample D (87.1 mA/mg_Ag_) compared to samples B and E highlights the excellent performance of this type of GDE even at very low mass-loading (Figure 4c).

As already mentioned, the structural characteristics of the carbon composite electrode are crucial in the determination of the catalytic behavior of the GDE in bicarbonate electrolyzers. In particular, the choice of the GDL is critical, as its permeability to the bicarbonate solution directly impacts the *i*-CO_2_ production efficiency. It is known that the catalyst’s selectivity toward CO tends to increase when the system is more proficient in producing *i*-CO_2_ [24].

**Figure 5:**
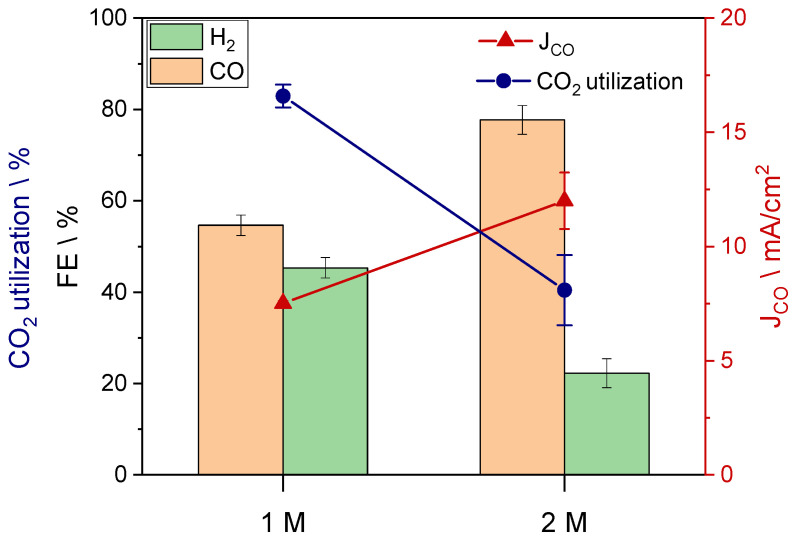
FE, CO_2_ utilization, and CO partial current density when varying the concentration of the bicarbonate solution. GDE sample: B.

This observation was further confirmed by comparing the performance of the same GDE (sample B) using a less concentrated bicarbonate solution. When the concentration is halved from 2 M to 1 M, the carbon feedstock is poorer and the *i*-CO_2_ generated drops. This introduces a mass transport limitation, causing a decrease in FE_CO_ from 77% to 55%, while the CO_2_ utilization drastically increased from 40% to 83%, as reported in Figure 5.

**Figure 6:**
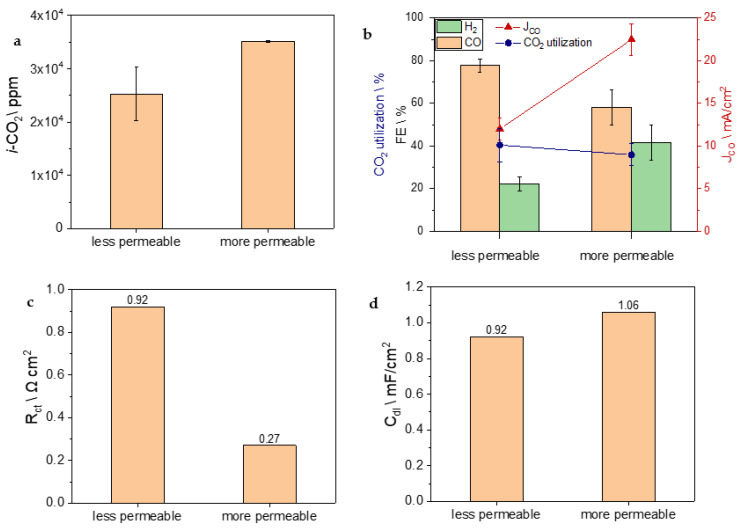
(**a**) Concentration of *i*-CO_2_ produced by the electrolyzer using carbon supports with different permeability: less permeable (sample B) and more permeable (sample F). (**b**) FE, CO_2_ utilization, and partial current density for CO. (**c**) Charge transfer resistance values and (**d**) double-layer capacitance values obtained by EIS analysis.

Increasing the permeability of the GDL would have a similar effect to using a higher electrolyte concentration. This improvement allows for enhanced flow of bicarbonate through the GDE, reaching the BPM, and consequently, the low-pH region becomes capable of producing a larger amount of *i*-CO_2_. Therefore, sample B was compared, whilst keeping the same mass loading (565 μg/cm^2^), to a GDE (sample F) whose GDL has a permeability that is four times higher. The effect of permeability is evident in Figure 6a, which shows the amount of CO_2_ released inside the reactor as a function of the GDE’s permeability. The graph presents the total *i*-CO_2_ produced, which was calculated by summing the concentrations of CO_2_ and CO detected at the electrolyzer outlet during electrolysis.

However, as shown in Figure 6b, the improvement in *i*-CO_2_ production given by the high permeability of sample F did not provide an enhancement of selectivity; in fact, FE_CO_ dropped from 77% in sample B to 58%. Most probably, having a very open structure (see FESEM micrographs in Figure S4), which allows it to be more permeable to the bicarbonate, introduces a problem of mass transportation of *i*-CO_2_ toward the active sites, affecting the FE_CO_. The R_ct_ provided by the EIS analysis in sample F is around three times lower (Figure 6c), meaning that it includes a higher number of active sites for catalysis, either for CO_2_RR or HER, as evidenced by the double-layer capacitance and displayed in Figure 6d. The slightly larger value of the C_dl_ of the most permeable GDE, 1.06 mF/cm^2^ compared to 0.92 mF/cm^2^, indicates a higher ECSA and confirms the presence of a larger number of active sites. The R_ct_ and C_dl_ values account for the high values of J_tot_ (Figure S1a), partial mass activity (Figure S1b), and J_CO_ observed in sample F, ensuring a good CO_2_ utilization percentage even with a lower FE_CO_ and increased *i*-CO_2_ production. In fact, the partial mass activity for CO was found to be 40 mA/mg_Ag_ (Figure S1b), surpassing values reported in the literature (Table 1). It is important to emphasize that despite the lower Faradaic efficiency, the significantly high J_CO_ achieved, explained by the higher ECSA, makes sample F likely the most suitable GDE for industrial purposes in syngas production.

## 4. Conclusions

In this work, novel high-performance Ag electrodes for bicarbonate electrolyzers were fabricated via a simple and scalable sputtering method. Silver thin films were deposited on commercial carbon supports and used as free-standing gas diffusion electrodes without any post-treatment. Thanks to the highly repeatable deposition technique, GDEs with different carbon substrates and silver mass loadings were reliably tested at V_cell_ = 3 V to understand their effect in terms of activity and selectivity in CO_2_-to-CO conversion. The final result of this investigation presents Ag-GDEs with a FE_CO_ close to 80%, which is comparable to the state-of-the-art achievement with a mass loading of 565 μg/cm^2^ (sample B). This mass loading is significantly lower compared to the well-performing Ag-GDEs reported in the literature. Moreover, increasing the permeability of the carbon GDL significantly enhanced the activity and, consequently, the mass-activity. As a result, sample F exhibited remarkably high partial mass activity compared to the values reported in the literature for bicarbonate electrolyzers. The new Ag electrode reported respectable results in terms of CO_2_ utilization, which turned out to be around 40%, while, when the bicarbonate concentration was halved to 1 M, it reached 83%. Additionally, our research marks a significant advancement in the field of GDE development for bicarbonate electrolyzers by introducing the application of electrochemical impedance spectroscopy. This innovative technique provided us with a valuable opportunity to delve deeper into the underlying factors that influenced the GDE’s performance within the reactor. In fact, the charge transport resistances and the double-layer capacitances derived from the fitting of the experimental Nyquist plot provided an effective explanation for the different behaviors of the GDEs during bicarbonate electrolysis. Therefore, by using EIS as a powerful characterization tool for GDEs in bicarbonate electrolyzers, this work contributes to the growing body of knowledge in this emerging field of research.

The profound insights gained from this study offer a comprehensive understanding of the intricate electrochemical processes taking place within GDEs during bicarbonate electrolysis. Based on the results obtained by this work, the herein-proposed Ag GDEs demonstrate exceptionally promising potential for low-cost electrodes in the future industrial implementation of integrated carbon capture and conversion through bicarbonate electrolyzers.

## Figures and Tables

**Table 1:** The reported state-of-the-art silver GDEs’ performance for liquid-fed bicarbonate electrolyzers.

Ag Mass Loading (mg/cm^2^)	Deposition Technique	Feedstock [KHCO_3_ (M)]	FE_CO_ (%)	Cell Potential (V)	J_CO_ (mA/cm^2^)	Partial Mass Activity (mA/mg_Ag_)	Reference
13 *	Spray coating	3	80	3	20	2 *	[7]
2	PVD + spray coating	3	25	3.5	25	13	[8]
2	Spray coating	2	58	3	14	7	[14]
Foam **	Free standing electrode **	3	60	3.7	60	-	[15]
3	Electrodeposition	3	70	3.5	70	23	[16]
2	PVD + spray coating	3	82	3.6	82	41	[17]
0.565	PVD	2	77	3	13	25	This work
0.565	PVD	2	58	3	22	40	This work
0.116	PVD	2	55	3	6	48	This work

* This is the nominal loading; the experimental one was not reported by T. Li et al. [7]. ** Loading not present since a silver foam was used as a free-standing GDE.

## Data Availability

Data are available upon request.

## Supplementary Materials

The following supporting information can be downloaded at: https://www.mdpi.com/article/10.3390/nano13162314/s1. Tables S1 and S2 on the features of commercial carbon papers and Ag-GDEs; Figure S1 reporting the total density current and the partial mass activity; details on the reactor set-up; formulas used to calculate Faradaic efficiencies, partial density current, *i*-CO_2_, CO_2_ utilization, mass activity, and partial mass activity; details on and results (Table S3 and Figure S2) of the EIS analysis; Figure S3 reports the values of J_tot_/C_dl_; Figure S4 displays the FESEM images of the GDEs. Ref. [25] is cited in the Supplementary Materials.
