# Supplementary material for: Optimizing the Performance of Low-Loaded Electrodes for CO2-to-CO Conversion Directly from Capture Medium: A Comprehensive Parameter Analysis
Source: Nanomaterials (Basel). 2023 Aug 12;13(16):2314. doi: 10.3390/nano13162314 (PMC10458350; doi:10.3390/nano13162314)
Supplement: Supplementary file 1 [file nanomaterials-13-02314-s001.zip › nanomaterials-2520019-supplementary.pdf]

nanomaterials

MDPI

Supporting Materials

Optimizing the Performance of Low-Loaded Electrodes for CO_2-to-CO Conversion Directly from Capture Medium: A Comprehensive Parameter Analysis

Alessio Mezza [1,2,*], Mattia Bartoli [1], Angelica Chiodoni [1], Juqin Zeng [1,2], Candido F. Pirri [1,2] and Adriano Sacco [1,*]

[1] Center for Sustainable Future Technologies @Polito, Istituto Italiano di Tecnologia, Via Livorno 60, 10144 Torino, Italy; mattia.bartoli@iit.it (M.B.); angelica.chiodoni@iit.it (A.C.); juqin.zeng@polito.it (J.Z.); fabrizio.pirri@polito.it (C.F.P.)

[2] Department of Applied Science and Technology, Politecnico di Torino, Corso Duca degli Abruzzi 24, 10129 Torino, Italy

* Correspondence: alessio.mezza@polito.it (A.M.); adriano.sacco@iit.it (A.S.)

Electrochemical testing

Flow Cell Design

A commercial electrolyzer cell (Cell Fixture, Scribner), whose schematic representation is reported in Fig.1, was used to carry out the bicarbonate electrolysis. It consisted of housing gaskets, two flow-field plates, two gold current collectors, and a membrane electrode assembly (MEA). The anode and cathode graphite flow field (active area: 5 cm^2) delivered a liquid feed through serpentine channels. The MEA placed between the flow fields consisted of a nickel foam (5 cm^2, thickness: 1.6 mm), a bipolar membrane, and a Ag/C GDE. The nickel foam was housed in a compressible ethylene propylene diene monomer (EPDM) with a nominal thickness of 1.6 mm, while the Ag/C cathode was contained in a 0.127 mm-thick polytetrafluoroethylene (PTFE) gasket. The current collectors, flow fields, and MEA were sandwiched by two stainless steel plates with eight bolts tightened to a torque of 11.3 N m.

Formulas

The faradaic efficiency (FE) was calculated based on gas products only using the equation below:

$$FE = \frac{V \cdot t \cdot C \cdot n \cdot F}{\mathrm{Vm} \cdot \mathrm{Q}} \quad \text{(S1)}$$

where V_m is the molar volume of an ideal gas (L mol^{-1}), V is the flow rate of the stream measured at the outlet of the cathodic side (L min^{-1}), Q is the total charge passed through the system during the electrolysis time t (C), C is the concentration of the gas product (% v/v), n is the number of electrons required to obtain one molecule of this product (n = 2 for CO and H_2 formation), and F is the faraday constant (96485 C mol^{-1}).

The partial density current (J_{CO}) was calculated by multiplying the total density current J_{tot} by the faradaic efficiency for CO:

$$J_{CO} = FE_{CO} \cdot J_{tot} \quad \text{(S2)}$$

The in situ generated CO_2 (*i*-CO_2) was calculated assuming that CO was the only reduced carbon product since a Ag electrocatalyst was used:

$$i\text{-}CO_2 = [CO_2]_{\mu GC} + [CO]_{\mu GC} \quad \text{(S3)}$$

where $[CO_2]_{\mu GC}$ and $[CO]_{\mu GC}$ are the concentrations of CO_2 and CO measured by the GC, respectively.

CO_2 utilization was calculated by dividing the concentration of CO at the outlet by the total amount of *i*-CO_2 produced:

$$\text{CO2 utilization} = \frac{[\text{CO}]\mu\text{GC}}{\text{i} - \text{CO2}} \quad (S4)$$

The mass activity was calculated by dividing the J_{tot} by the silver mass loading:

$$\text{activity} = \frac{\text{Jtot mass activity}}{\text{mass loading}} \quad (S5)$$

where the mass loading was obtained by weighing the sample before and after silver deposition and by dividing the difference in weights by the geometric area of the GDL.

The partial mass activity was calculated by multiplying the mass activity by the faradaic efficiency for CO:

$$\text{partial mass activity} = \text{mass activity} \cdot FE_{CO} \quad (S6)$$

Electrochemical Impedance Spectroscopy analysis

Representative Nyquist plots of the impedance related to the Ag-based B, D, E, and F GDE samples acquired at –1 V are reported in Figure S2. The experimental EIS data were fitted through an equivalent circuit (inset of Figure S2), comprising a series resistance (accounting for the electrolyte conductivity) and two resistance/capacitance parallels, accounting for the high- (charge transport) and low-frequency (charge transfer) processes [1]. The values of such parameters are shown in Table S3.

Table S1. Summary of the characteristics of the GDLs Sigracet (ion power) used to prepare the Ag GDEs (ref: https://www.fuelcellstore.com/spec-sheets/sigracet-gdl-white-paper-newgeneration.pdf).

Carbon paper code	PTFE	MPL	Thickness (μm)	Permeability ($10^{-12} m^2$)
28 AA			190	2 – 3
28 BC	√	√	235	1.4
29 AA			190	8 - 9

Table S2. Summary of the composition of all of the GDEs tested in this work.

Sample	Carbon support	Sputtering time	Sputtered faces	Silver mass – loading
A	28 AA	300 s	1	282 μg / cm^6
B	28 AA	300 s	2	565 μg / cm^6
C	28 BC	300 s	2	565 μg / cm^6
D	28 AA	100 s	2	116 μg / cm^6
E	28 AA	600 s	2	1.13 mg / cm^6
F	29 AA	300 s	2	656 μg / cm^6

Table S3. Summary of the values of the fitting parameters obtained from the EIS analysis.

Sample	$R_s\ (\Omega \cdot cm^2)$	$R_t\ (\Omega \cdot cm^2)$	$R_{ct}\ (\Omega \cdot cm^2)$	$C_t\ (\mu F / cm^2)$	$C_{dl}\ (\mu F / cm^2)$
B	1.65	0.08	0.92	1311	0.92
D	2.78	0.07	1.42	712	1.01
E	1.27	0.1	0.38	113	1.51
F	0.83	0.39	0.27	7.39	1.06

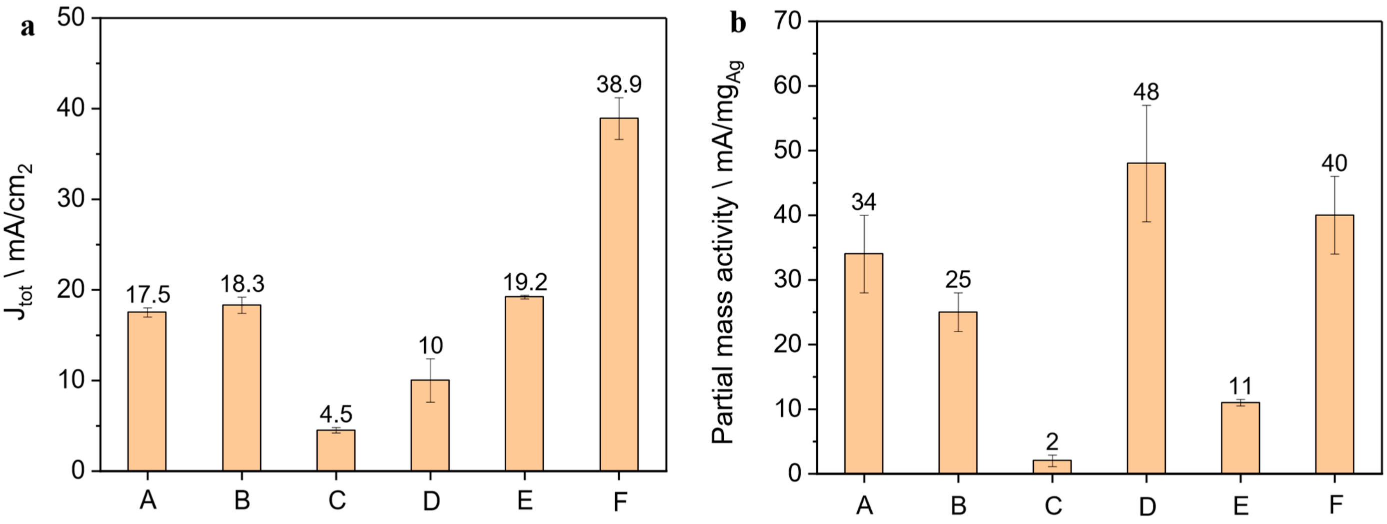

Figure S1. (a) Activities and (b) partial mass activities obtained by testing all of the GDEs under the same conditions in the bicarbonate electrolyzer by applying $V_{cell} = 3\ V$.

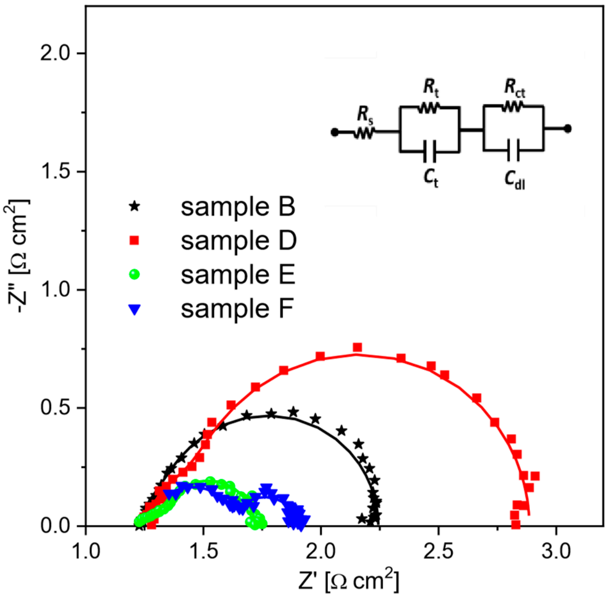

Figure S2. Impedance spectra of the different Ag-GDEs. The points are the experimental data and the continuous lines are the curves calculated through a fitting procedure using the equivalent circuit shown in the inset.

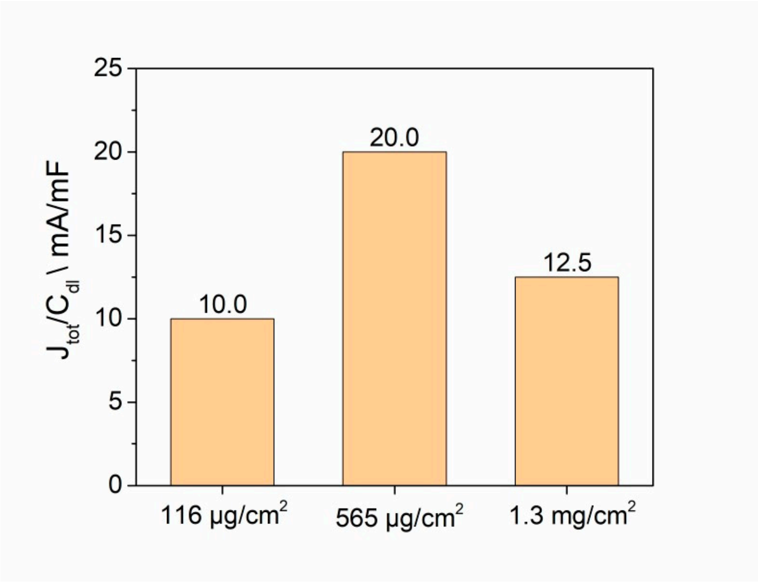

Figure S3. Values of C_{dl} normalized current densities (J_{tot}/C_{dl}) for sample with different catalyst mass loadings.

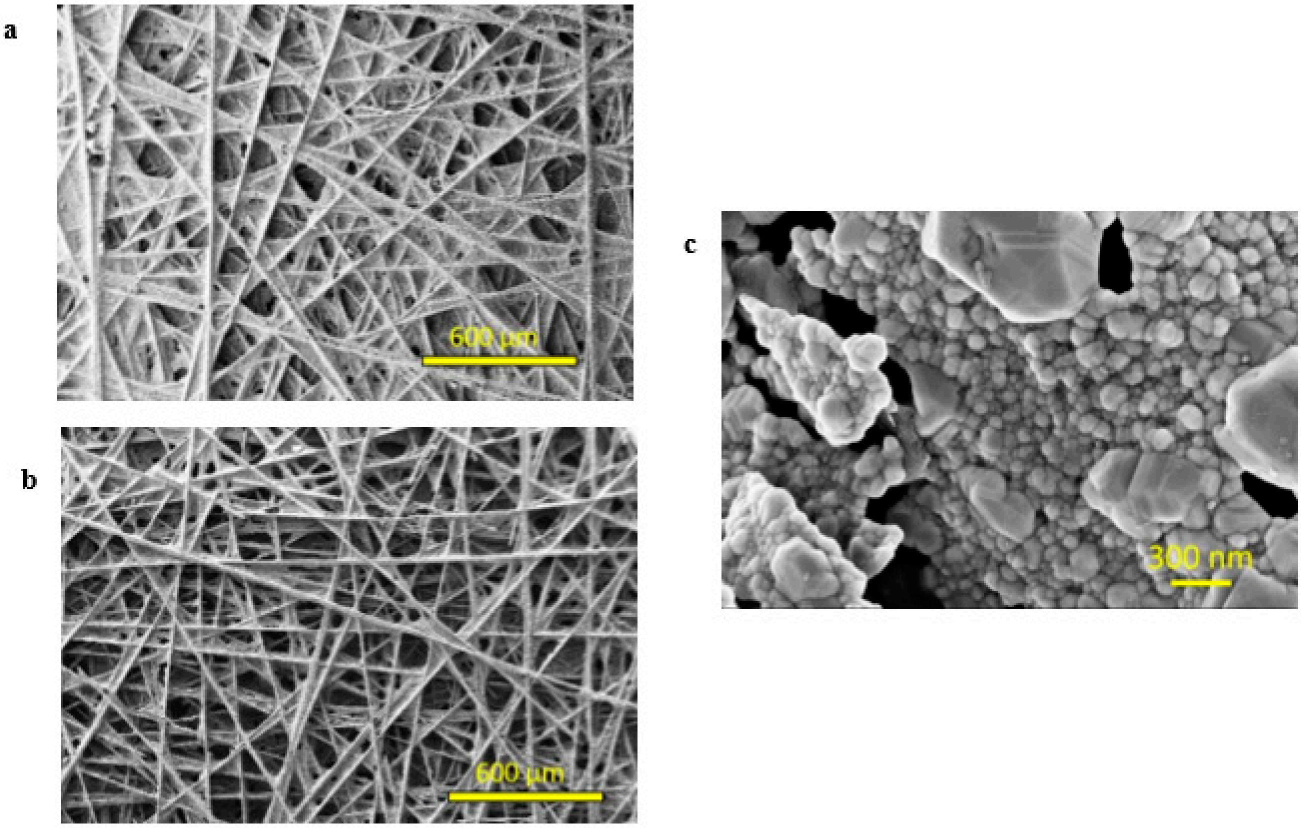

Figure S4. Arrangement of the carbon fiber of the commercial carbon supports: **(a)** Sigracet 28 AA, **(b)** Sigracet 29 AA. **(c)** Silver nanoparticles grown on the carbon support after the sputtering deposition.

REFERENCES

[1] Zeng J, Rino T, Bejtka K, Castellino M, Sacco A, Farkhondehfal MA, et al. Coupled Copper– Zinc Catalysts for Electrochemical Reduction of Carbon Dioxide. 2020;13:4128-39.
